# Use of mHealth Devices to Screen for Atrial Fibrillation: Cost-Effectiveness Analysis

**DOI:** 10.2196/20496

**Published:** 2020-10-06

**Authors:** Godwin D Giebel

**Affiliations:** 1 E-Government/E-Health, Department of Business Information Systems Baden-Wuerttemberg Cooperative State University Mannheim Mannheim Germany

**Keywords:** mHealth, atrial fibrillation, screening devices, strokes, cost-effectiveness, photoplethysmography

## Abstract

**Background:**

With an estimated prevalence of around 3% and an about 2.5-fold increased risk of stroke, atrial fibrillation (AF) is a serious threat for patients and a high economic burden for health care systems all over the world. Patients with AF could benefit from screening through mobile health (mHealth) devices. Thus, an early diagnosis is possible with mHealth devices, and the risk for stroke can be markedly reduced by using anticoagulation therapy.

**Objective:**

The aim of this work was to assess the cost-effectiveness of algorithm-based screening for AF with the aid of photoplethysmography wrist-worn mHealth devices. Even if prevented strokes and prevented deaths from stroke are the most relevant patient outcomes, direct costs were defined as the primary outcome.

**Methods:**

A Monte Carlo simulation was conducted based on a developed state-transition model; 30,000 patients for each CHA_2_DS_2_-VASc (Congestive heart failure, Hypertension, Age≥75 years, Diabetes mellitus, Stroke, Vascular disease, Age 65-74 years, Sex category [female]) score from 1 to 9 were simulated. The first simulation served to estimate the economic burden of AF without the use of mHealth devices. The second simulation served to simulate the economic burden of AF with the use of mHealth devices. Afterwards, the groups were compared in terms of costs, prevented strokes, and deaths from strokes.

**Results:**

The CHA_2_DS_2_-VASc score as well as the electrocardiography (ECG) confirmation rate had the biggest impact on costs as well as number of strokes. The higher the risk score, the lower were the costs per prevented stroke. Higher ECG confirmation rates intensified this effect. The effect was not seen in groups with lower risk scores. Over 10 years, the use of mHealth (assuming a 75% ECG confirmation rate) resulted in additional costs (€1=US $1.12) of €441, €567, €536, €520, €606, €625, €623, €692, and €847 per patient for a CHA_2_DS_2_-VASc score of 1 to 9, respectively. The number of prevented strokes tended to be higher in groups with high risk for stroke. Higher ECG confirmation rates led to higher numbers of prevented strokes. The use of mHealth (assuming a 75% ECG confirmation rate) resulted in 25 (7), –68 (–54), 98 (–5), 266 (182), 346 (271), 642 (440), 722 (599), 1111 (815), and 1116 (928) prevented strokes (fatal) for CHA_2_DS_2_-VASc score of 1 to 9, respectively. Higher device accuracy in terms of sensitivity led to even more prevented fatal strokes.

**Conclusions:**

The use of mHealth devices to screen for AF leads to increased costs but also a reduction in the incidence of stroke. In particular, in patients with high CHA_2_DS_2_-VASc scores, the risk for stroke and death from stroke can be markedly reduced.

## Introduction

With an estimated prevalence of about 3%, atrial fibrillation (AF) is one of the most common cardiac arrhythmias [1]. On the one hand, AF can be considered as an independent disease; on the other hand, AF can be considered as a risk factor for secondary diseases. AF is associated with an increased risk of all-cause mortality, as well as cardiovascular mortality and stroke [2,3].

An established way to estimate the risk for stroke in patients with AF is the CHA_2_DS_2_-VASc score (Congestive heart failure, Hypertension, Age≥75 years, Diabetes mellitus, Stroke, Vascular disease, Age 65-74 years, Sex category [female]) [4]. To reduce the risk of stroke, it is recommended to consider anticoagulation therapy after the diagnosis of AF in male patients with a CHA_2_DS_2_-VASc score of 1 and in female patients with a score of 2 [1].

AF can occur in 5 different forms (first diagnosed, paroxysmal, persistent, long-standing persistent, and permanent), which can be either symptomatic or asymptomatic. The European Society of Cardiology recommends opportunistic screening by pulse taking or electrocardiogram rhythm strip in patients older than 65 years because undiagnosed AF remains a common problem [1].

While screening during visits to the doctor often misses irregular forms of AF, screening with the aid of implantable cardioverter-defibrillators, pacemakers, and implantable loop recorders is, at the same time, only eligible for a minority of patients with previous cardiac illnesses. An innovative and accurate approach to detect AF might be the application of mobile health (mHealth) in combination with algorithms. Nevertheless, the diagnosis should always be confirmed by electrocardiography (ECG) as the gold standard [1].

The aim of this work was to evaluate the fictitious use of photoplethysmography (PPG) in combination with algorithms integrated in wrist-worn mHealth devices over a period of 10 years to support the diagnosis of AF as an add-on to the existing health care system in Germany. The focus of this study was on the different outcomes. The primary outcome was AF-related costs. The secondary outcomes were the number of prevented strokes and prevented deaths from stroke.

## Methods

### Model Description

**Figure 1:**
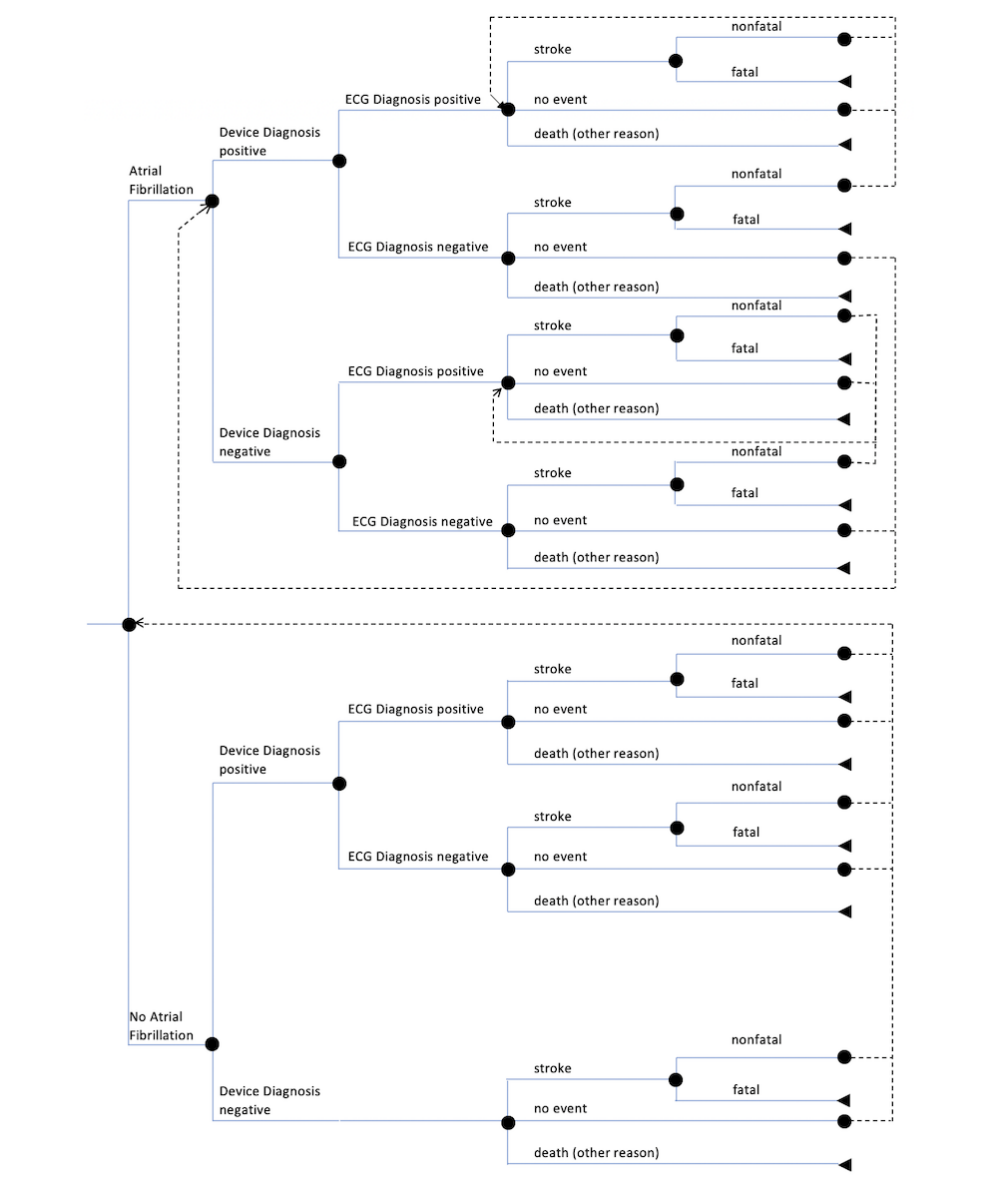
Model structure of the group with mobile health devices (each end point is a different scenario). Additional bleeding events can occur in each end point. ECG: electrocardiography.

**Figure 2:**
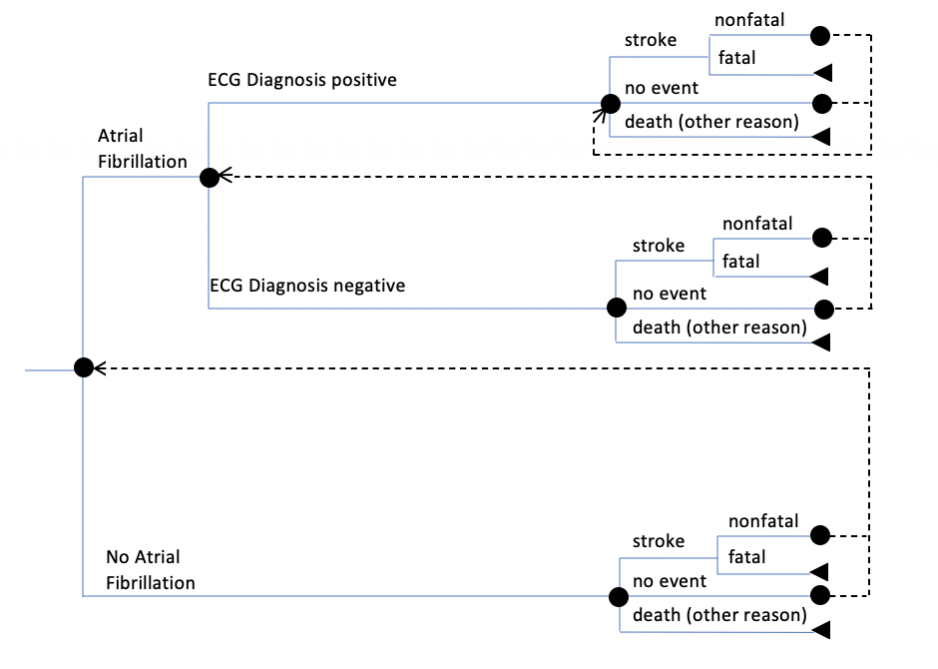
Model structure of the group without mobile health devices (each end point is a different scenario). Additional bleeding events can occur in each end point. ECG: electrocardiography.

A Markov Model, a practical tool for medical decision making [5], was developed to assess the health economic impact of wrist-worn PPG mHealth devices in the diagnosis of AF. A model previously published by Reinhold et al who compared implantable cardioverter-defibrillators was adapted [6]. A Monte Carlo simulation was conducted based on a developed state-transition model. Depending on the underlying patient group, either with or without devices, different states and transitions were restricted (Figure 1 and Figure 2). For both groups, simulations were based on a time horizon of 10 years. This was assumed since technological changes might probably lead to even more accurate devices. During this period, changings of state were calculated based on a 1-year cycle. Whether the health state of individuals changes or not, depends on the previous state as well as on defined probabilities of state transition as listed in Table 1.

The simulation ends for an individual in case of death or by reaching the time horizon of 10 years. In all other cases, the subject re-enters the simulation at a point defined by the previous state. The re-entering points are indicated in Figure 1 and Figure 2. The end point of a 1-year cycle is the starting point for the next cycle.

An individual enters the simulation either with AF or without AF. The initial health state is defined by the prevalence of AF. The following path is determined by the incidence of the alternatives at each decision node. Cardioversion through surgical interventions (eg, catheter ablation) to restore normal sinus rhythm was excluded. Thus, it was assumed that once an individual experiences AF, it cannot be cured. With AF, an individual cannot leave the upper branch (Figure 1 and Figure 2, “Atrial Fibrillation”) of the decision tree.

**Table 1:** Probabilities of annual state transition as well as underlying assumptions and sources.

Serial number	Model item	Assumptions	Sources and description
1	Prevalence of AF^a^ at baseline	Based on the CHA_2_DS_2_-VASc^b^ score: 0.01, 0.015, 0.034, 0.067, 0.118, 0.182, 0.255, 0.302, 0.403, 0.492	Derived from the study of Saliba et al [7]. The prevalence was used to simulate the initial proportion of patients with AF.
2	Incidence of AF in the general population (per 100 person-years)	Based on the CHA_2_DS_2_-VASc score: 0.17, 0.21, 0.49, 0.94, 1.65, 2.31, 2.75, 3.39, 4.09, 6.71	Derived from the study of Saliba et al [7]. The incidence was used to estimate the number of new cases of AF each year.
3	Sensitivity of mHealth^c^ devices	93%^d^	Derived from the study of Bonomi et al [8]. Sensitivity of PPG compared to 24/48-hour Holter electrocardiogram readings in outpatient settings; 93 out of 100 patients with AF receive a true-positive diagnosis.
4	False-positive AF detection rate (mHealth device)	0.2%^d^	Bonomi et al [8] described the false-positive detection rate as lower than 0.2%; 0.2% of subjects without AF receive a false-positive diagnosis.
5	Confirmation of the mHealth diagnosis (by a physician using ECG^e^)	100%, 75%, and 50%	Because of the nonpersistent forms of AF, the disease cannot always be confirmed through ECG follow-up. Nevertheless, in the first step, the assumption was made that a true-positive mHealth diagnosis of AF can always be confirmed by a physician. In subsequent simulations, the proportion was altered.
6	Clarification of a wrong mHealth diagnosis (by a physician using ECG)	100%	Assumption that in patients with no AF, the attending physician will not find artefacts of arrhythmia in the electrocardiogram.
7	Proportion of AF detected without a device	36.09%	Steinhubl et al [9] investigated the detection rate of AF in active home-based monitored individuals. They found newly diagnosed AF in 6.7 per 100 person-years in the monitored individuals and 2.6 per 100 person-years in unmonitored individuals. The proportion of AF detected with the aid of wearables was multiplied with the AF ratio between unmonitored and monitored individuals. Yearly, 36.09% of AF cases can be detected without the use of mHealth devices.
8	Stroke incidence in untreated patients with AF (per 100 person-years)	Based on the CHA_2_DS_2_-VASc score: 0.2, 0.6, 2.5, 3.7, 5.5, 8.4, 11.4, 13.1, 12.6, 14.44	Derived from the study of Friberg et al [10]. The stroke incidence yields the probability of experiencing a stroke.
9	Stroke incidence in patients with no AF (per 100 person-years)	Based on the CHA_2_DS_2_-VASc score: 0.0826, 0.2479, 1.0331, 1.5289, 2.2727, 3.4711, 4.7107, 5.4132, 5.2066, 5.9669	According to Odutayo et al [2], patients with AF have a 2.42-fold increased risk for stroke compared to patients with no AF. The stroke incidence in untreated patients with AF was divided by 2.42.
10	Stroke incidence in patients with AF receiving NOAC^f^ (per 100 person-years)	Based on the CHA_2_DS_2_-VASc score: 0.068, 0.204, 0.85, 1.258, 1.87, 2.856, 3.876, 4.454, 4.284, 4.9096	VKA^g^ reduces the risk of stroke by two-third (66%) [1]. Rivaroxaban is noninferior to warfarin [11]. Thus, the risk reduction through NOAC should be at least as high as the one from VKA.
11	Stroke mortality in patients with no AF	34%	Derived from the study of Reinhold et al [6]. If a patient does not have AF but experiences a stroke, there is a 34% probability that the stroke is fatal.
12	Stroke mortality in untreated patients with AF	63%	Derived from the study of Reinhold et al [6]. If a patient has AF and does not receive medication, there is a 63% probability that the stroke is fatal.
13	Stroke mortality in patients with AF receiving NOAC	42%	Derived from the study of Reinhold et al [6]. If a patient has AF and receives medication, the probability that an occurring stroke is fatal is 42%.
14	Mortality in patients with no AF, no stroke	6%	Derived from the study of Reinhold et al [6]. Probability that an individual who does not have AF dies due to reasons other than stroke.
15	Mortality in untreated patients with AF, no stroke	11.1%	Derived from the study of Reinhold et al [6]. The probability that an untreated patient with AF dies due to reasons other than stroke.

^a^AF: atrial fibrillation.

^b^CHA_2_DS_2_-VASc: Congestive heart failure, Hypertension, Age≥75 years, Diabetes mellitus, Stroke, Vascular disease, Age 65-74 years, Sex category (female).

^c^mHealth: mobile health.

^d^values were changed in sensitivity analysis.

^e^ECG: electrocardiography.

^f^NOAC: non–vitamin K antagonist.

^g^VKA: vitamin K antagonist.

Once the individual health state is set and the underlying individual is part of the group with mHealth devices, there is a given probability of a device-based diagnosis (either true-positive diagnosis or false-positive diagnosis). If the device-based diagnosis is positive, the patient visits a doctor and an ECG is recorded. If the mHealth diagnosis was false positive, the doctor will clear up the misdiagnosis and the individual is considered as healthy and remains in the group without AF. If the individual truly has AF and the mHealth device–based diagnosis is positive, the diagnosis might be confirmed by the doctor. Either way, if the diagnosis is confirmed or not, the patient remains in the AF group (Figure 1). Therefore, different probabilities were assumed (Table 1, Serial number 5).

In case the device misses a diagnosis of AF or the individual is in the group without mHealth devices, there is a chance that AF is diagnosed during a visit to the physician in terms of standard care (Table 1, Serial number 7). Furthermore, it is supposed that a stroke in patients with previously undetected AF leads to an AF diagnosis and therapy as well.

Once an individual receives an ECG-driven diagnosis of AF, it is valid for the rest of the simulation and the possible states are restricted according to the state-transition model. Based on the diagnosis, it is assumed that the patient receives anticoagulation therapy in the form of non–vitamin K antagonists (NOAC).

The possible end points at the end of each cycle are identical, irrespective of the preceding arms of the decision tree. The first possible end point could be experiencing a stroke, which can be either fatal or nonfatal. The second possible end point could be that the individual does not face any event influencing the simulation. The third end point could be that the patient can die due to reasons other than stroke. In all the end points, additional bleeding events can occur.

### State Transition Probabilities

The underlying probabilities for state transition are depicted in Table 1. The transition possibilities differ for the implemented CHA_2_DS_2_-VASc score. Increasing scores correlate with higher prevalence and incidence of AF as well as higher risk for stroke. The initiation of NOAC reduces the risk of stroke and mortality in patients with AF; however, it increases the risk for major bleeding. To assess the accuracy of mHealth devices in screening for AF, a study focusing on the use of PPG was used [8]. PPG is one of the most widespread technologies in mHealth devices to screen for AF.

### Costs

AF-related direct costs were considered from the view of the German statutory health insurance. Device costs, costs incurred during a visit to the doctor, costs incurred in diagnostics, costs incurred in the therapy in form of NOAC, as well as costs related to stroke and major bleeding were integrated. Device costs were derived from the most popular mHealth AF screening device, the Apple Watch 5 (€437.65, €1=US $1.12) [12].

**Figure 3:**
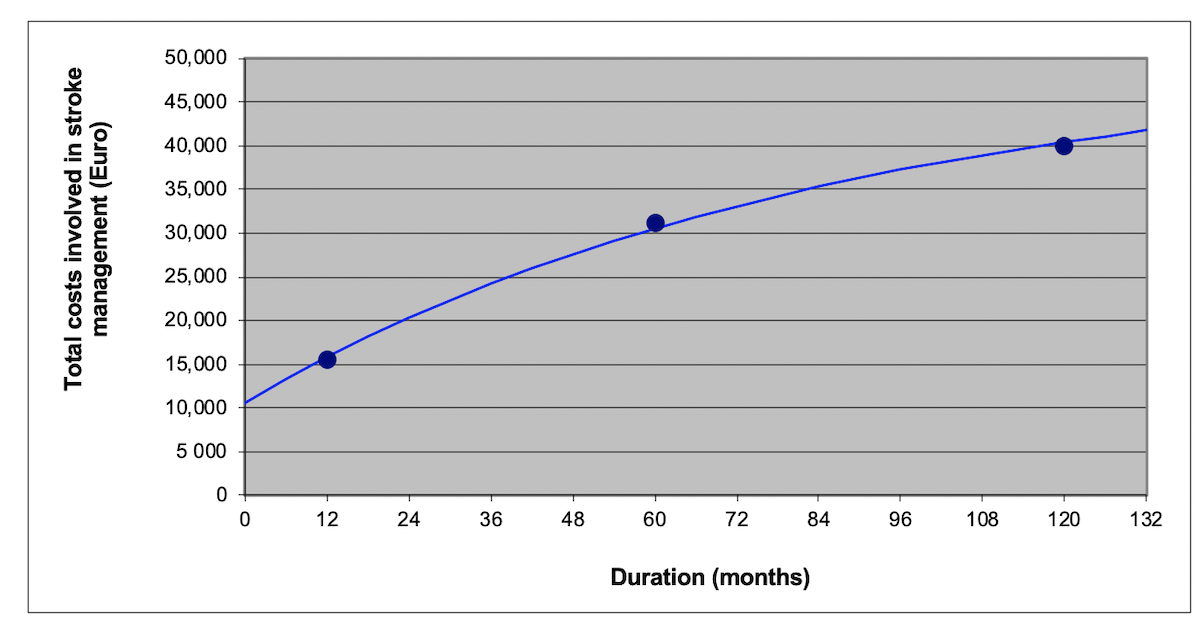
Interpolation and extrapolation of costs determined by using least squares adjustment. Values for year 1, year 5, and year 10 derived from Kolominsky-Rabas et al [16]. €1=US $1.12.

To confirm the mHealth device–based diagnosis by a physician, the costs were represented by adding single cost factors incurred during the physician visit (ordination, consultation, urgent care, telephone advice, telemedical care) (€35.62) with cost factors resulting from diagnostics (long-term ECG, 12-lead ECG, stress ECG) (€31.61) [13,14]. The cost components were derived from [13] but the costs were adapted to the year of the study. As medication costs for oral anticoagulation, the use of rivaroxaban as the most prescribed NOAC in Germany was assumed. Thus, the costs for pharmaceuticals resulted in €1226 per year [15]. Costs for individuals with stroke, either fatal or not, were derived from the study of Kolominsky-Rabas et al [16]. An interpolation and an extrapolation were made to receive period-specific costs (Figure 3 and Table 2). The costs for major bleeding (€1995) were directly derived from the study of Reinhold et al [6]. The present value was calculated using a discount rate of 3% per year.

**Table 2:** Relevant cost factors as well as sources and descriptions.

Cost factor	Costs^a^	Reasons and description
Device costs	€437.65	The price was derived from the most popular PPG^b^ AF^c^ screening device, the Apple Watch Series 5 [12]. An integrated algorithm diagnoses AF automatically. Trained personnel for interpretation is not needed.
Visit to the doctor and diagnostics	€67.23	Physician visit: ordination and consultation, €13.20; urgent care, €12.90; and telemedical care, €9.52. Diagnostics: long-term ECG and 12-lead ECG, €9.96; stress ECG, €21.65; derived from the study of McBride et al [13] and adapted to current conditions [14].
Medication costs for oral anticoagulation (NOAC^d^)	€1226	The use of rivaroxaban was assumed because it is the most prescribed NOAC in Germany [15].^e^
Per year costs incurred after surviving a stroke	€15,753 (year 1), €4480 (year 2)… €1481 (year 10)	Interpolation and extrapolation of costs derived from the study of Kolominsky-Rabas et al [16] (Figure 3).^e^
Costs for major bleeding	€1995	Directly derived from the study of Reinhold et al [6].
Annual discounting rate	3%	Own assumption.

^a^€1=US $1.12.

^b^PPG: photoplethysmography.

^c^AF: atrial fibrillation.

^d^NOAC: non–vitamin K antagonist.

^e^The program was realized using unrounded amounts in Euro.

### Implementation

As relevant outcomes costs, prevented strokes and prevented deaths from stroke were defined. To receive these outcomes, an implementation of the simulation was conducted in Excel (Microsoft Corp) by using Visual Basic for Applications.

Four different scenarios were simulated for each CHA_2_DS_2_-VASc score from 1 to 9: 3 scenarios with mHealth devices but different ECG confirmation rates (100%, 75%. and 50%) (Table 1, Serial number 5) and 1 scenario for patients without mHealth devices. Each simulation included 30,000 fictitious patients. Subsequently, a sensitivity analysis for device sensitivity and false-positive AF detection rate was conducted. According to the European Society of Cardiology Guidelines for the management of AF, it was assumed that anticoagulation therapy was initiated in male patients with a CHA_2_DS_2_-VASc score of 1 and in female patients with a score of 2 [1]. Therefore, a comparison in patients with a risk score of 0 was deemed as dispensable. To estimate the difference in the patients with a risk score of 1, it was assumed that half of the individuals were females. This is in accordance with the distributions of the sexes in the publications used to determine the prevalence and incidence of AF [7] as well as the stroke incidence [10] used in the simulation.

## Results

### Costs

**Figure 4:**
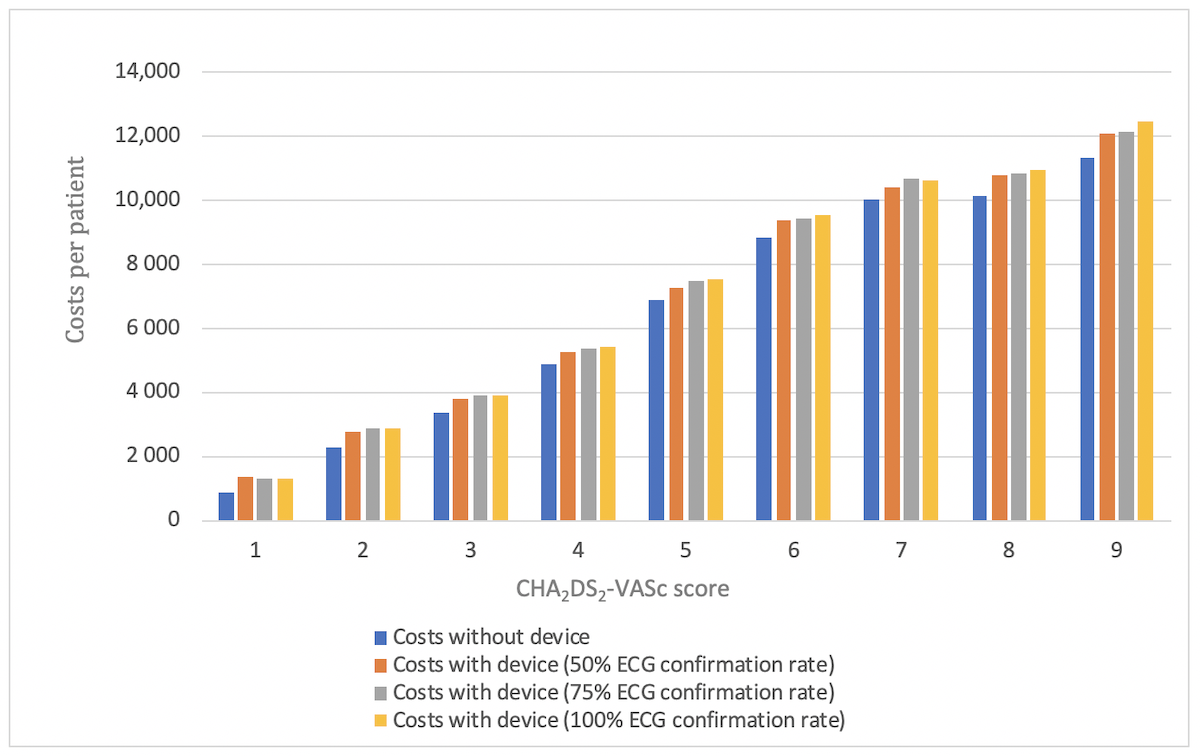
Costs per patient classified on the basis of the CHA_2_DS_2_-VASc score as well as the investigated group (with or without device and ECG confirmation rate). ECG: electrocardiography; CHA_2_DS_2_-VASc: Congestive heart failure, Hypertension, Age≥75 years, Diabetes mellitus, Stroke, Vascular disease, Age 65-74 years, Sex category (female). €1=US $1.12.

The economic effect of mHealth intervention was assessed in 2 steps. First, the focus was on costs per patient. Secondly, costs were assessed in relation to prevented strokes and fatal strokes. As seen in Table 3 and Table 4, an increasing risk score has a major impact on costs per patient in all the groups. The higher the CHA_2_DS_2_-VASc score, the higher are the costs per patient on average. While device ECG confirmation rate has little impact on costs per patient, the use of mHealth devices increases the costs per patient clearly (Figure 4).

To assess the costs per prevented stroke, the groups with and without mHealth devices were compared. The difference in the sum of the costs for all the patients in each group as well as the difference in the number of strokes were determined for each risk score. The ratio between the difference of the sum of costs and the difference in number of strokes resulted in costs per prevented stroke (Table 5).

Although costs per patient increase with increasing CHA_2_DS_2_-VASc scores, the costs per stroke tend to decrease in general. This effect is intensified by an increasing ECG confirmation rate. The effect is not seen in groups with lower risk scores. In these groups, the underlying basic risk for stroke is low. Thus, the risk reduction by use of mHealth devices is low as well. Findings for costs per fatal stroke fluctuated more than costs per patient and the number of fatal strokes. This can be explained by a small denominator (number of prevented [fatal] strokes) in relation to a large numerator (cost difference for all patients). Thus, small changes in the number of prevented (fatal) strokes have a big impact on costs per prevented (fatal) stroke.

With increasing ECG confirmation rates, the effect of mHealth use becomes more evident. Low ECG confirmation rates lead to results mainly driven by chance. In particular, regarding the costs per prevented fatal stroke, the impact of higher risk scores as well as ECG confirmation rates is even more pronounced.

**Table 3:** Summarized results of the simulations. Costs, strokes, and fatal strokes classified on the basis of the CHA_2_DS_2_-VASc score as well as the investigated group (N=30,000 patients per group per score).

	Study arm without device				Study arm with device (50% ECG^a^ confirmation)			
CHA_2_DS_2_-VASc score^b^	Average costs per patient (in €^c^, whole simulation duration)	Total number of strokes^d^	Number of nonfatal strokes	Number of fatal strokes	Average costs per patient (in €, whole simulation duration)	Total number of strokes^d^	Number of nonfatal strokes	Number of fatal strokes
1	873	581	379	202	1330	599	402	197
2	2280	2338	1571	767	2788	2351	1513	838
3	3351	3493	2283	1210	3815	3460	2232	1228
4	4860	5260	3288	1972	5239	4903	3100	1803
5	6877	7808	4844	2964	7233	7437	4569	2858
6	8802	10,397	6286	4111	9375	10,163	6228	3935
7	10,023	11,804	7024	4780	10,414	11,237	6857	4380
8	10,154	11,485	6591	4894	10,761	11,039	6469	4570
9	11,299	12,565	6944	5621	12,086	12,201	6964	5237
mean	6502	7303	4357	2947	7005	7043	4259	2784

^a^ECG: electrocardiography.

^b^CHA_2_DS_2_-VASc: Congestive heart failure, Hypertension, Age≥75 years, Diabetes mellitus, Stroke, Vascular disease, Age 65-74 years, Sex category (female).

^c^€1=US $1.12.

^d^Total number of strokes includes nonfatal and fatal strokes.

**Table 4:** Summarized results of the simulations. Costs, strokes, and fatal strokes classified on the basis of the CHA_2_DS_2_-VASc score as well as the investigated group (N=30,000 patients per group per score).

	Study arm with device (75% ECG^a^ confirmation)				Study arm with device (100% ECG confirmation)				
CHA_2_DS_2_-VASc score^b^	Average costs per patient (in €^c^, whole simulation duration)	Total number of strokes^d^	Number of nonfatal strokes	Number of fatal strokes		Average costs per patient (in €, whole simulation duration)	Total number of strokes^d^	Number of nonfatal strokes	Number of fatal strokes
1	1314	556	361	195		1290	528	331	197
2	2847	2406	1585	821		2876	2364	1550	814
3	3887	3395	2180	1215		3876	3339	2180	1159
4	5380	4994	3204	1790		5421	4894	3154	1740
5	7483	7444	4751	2693		7543	7263	4700	2563
6	9427	9755	6084	3671		9508	9549	6107	3442
7	10,646	11,082	6901	4181		10,627	10,703	6771	3932
8	10,846	10,374	6295	4079		10,937	10,122	6301	3821
9	12,146	11,449	6756	4693		12,463	11,210	6897	4313
mean	7108	6828	4235	2593		7171	6664	4221	2442

^a^ECG: electrocardiography.

^b^CHA_2_DS_2_-VASc: Congestive heart failure, Hypertension, Age≥75 years, Diabetes mellitus, Stroke, Vascular disease, Age 65-74 years, Sex category (female).

^c^€1=US $1.12.

^d^Total number of strokes includes nonfatal and fatal strokes.

**Table 5:** Number of prevented strokes and costs per prevented stroke in each intervention group.

Study arm, CHA_2_DS_2_-VASc score^a^		Cost difference for all patients^b^ (in €^c^)	Prevented strokes	Costs per prevented stroke (in €)	Prevented fatal strokes	Costs per prevented fatal stroke (in €)
**Study arm with device (100% ECG^d^ confirmation)**						
	1	12,519,300	53	236,213	5	2,503,860
	2	17,893,200	–26	–688,200	–47	–380,706
	3	15,759,300	154	102,333	51	309,006
	4	16,852,500	366	46,045	232	72,640
	5	19,992,600	545	36,684	401	49,857
	6	21,174,300	848	24,970	669	31,651
	7	18,103,800	1101	16,443	848	21,349
	8	23,481,300	1363	17,228	1073	21,884
	9	34,921,800	1355	25,773	1308	26,699
**Study arm with device (75% ECG confirmation)**						
	1	13,228,200	25	529,128	7	1,889,743
	2	17,028,300	–68	–250,416	–54	–315,339
	3	16,074,000	98	164,020	–5	–3,214,800
	4	15,609,900	266	58,684	182	85,769
	5	18,181,800	364	49,950	271	67,092
	6	18,732,600	642	29,179	440	42,574
	7	18,676,800	722	25,868	599	31,180
	8	20,762,700	1111	18,688	815	25,476
	9	25,423,200	1116	22,781	928	27,396
**Study arm with device (50% ECG confirmation)**						
	1	13,704,000	–18	–761,333	5	2,740,800
	2	15,242,700	–13	–1,172,515	–71	–214,686
	3	13,933,500	33	422,227	–18	–774,083
	4	11,367,300	357	31,841	169	67,262
	5	10,708,500	371	28,864	96	111,547
	6	17,187,900	234	73,453	176	97,659
	7	11,712,000	567	20,656	400	29,280
	8	18,208,800	446	40,827	324	56,200
	9	23,614,500	364	64,875	384	61,496

^a^CHA_2_DS_2_-VASc: Congestive heart failure, Hypertension, Age≥75 years, Diabetes mellitus, Stroke, Vascular disease, Age 65-74 years, Sex category (female).

^b^Cost difference between group with devices and group without devices.

^c^€1=US $1.12.

^d^ECG: electrocardiography.

### Prevented Strokes

**Figure 5:**
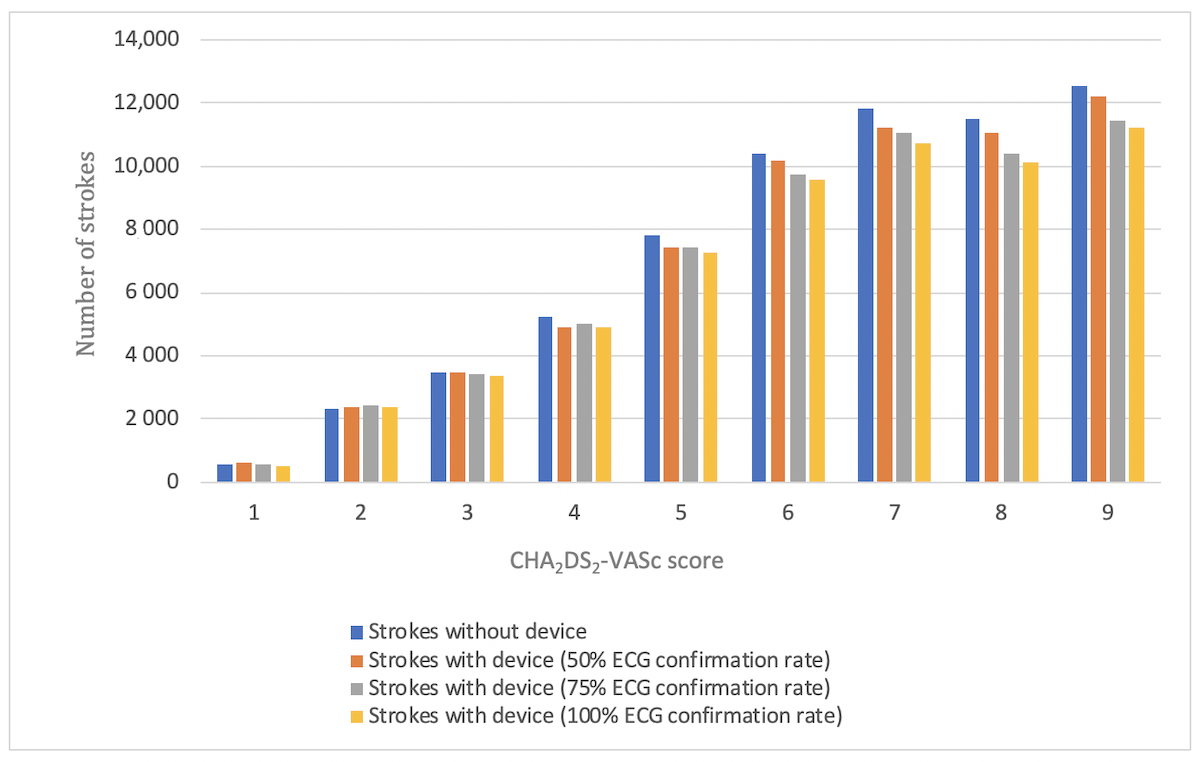
Stroke analysis on the basis of the CHA_2_DS_2_-VASc score as well as the investigated group (with or without device and ECG confirmation rate). ECG: electrocardiography; CHA_2_DS_2_-VASc: Congestive heart failure, Hypertension, Age≥75 years, Diabetes mellitus, Stroke, Vascular disease, Age 65-74 years, Sex category (female).

**Figure 6:**
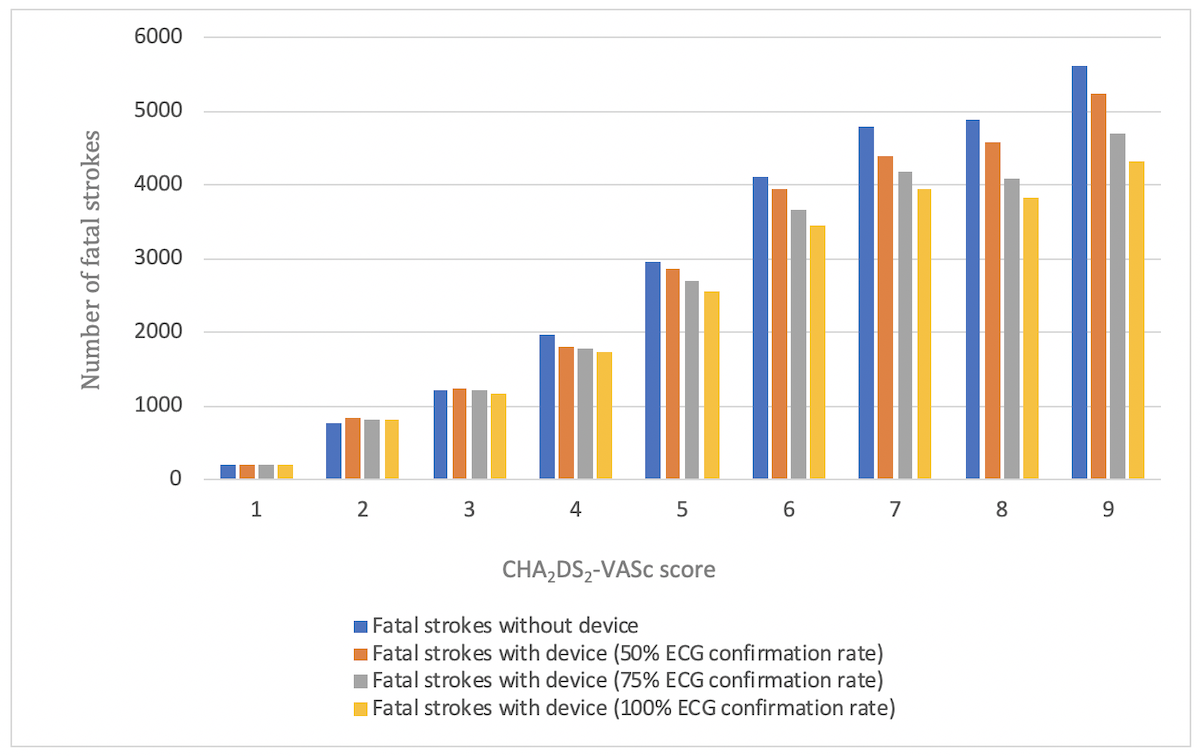
Fatal stroke analysis on the basis of the CHA_2_DS_2_-VASc score as well as the investigated group (with or without device and ECG confirmation rate). ECG: electrocardiography; CHA_2_DS_2_-VASc: Congestive heart failure, Hypertension, Age≥75 years, Diabetes mellitus, Stroke, Vascular disease, Age 65-74 years, Sex category (female).

With respect to patients, prevented strokes are considered as the most relevant outcome in this Monte Carlo simulation. Prevented strokes were analyzed as prevented strokes in total on the one hand and as prevented fatal strokes on the other hand. Both of them were calculated as the difference between the number of (fatal) strokes in the group without devices and the number of (fatal) strokes in each of the groups with devices (Table 5, Figure 5, and Figure 6).

The chance to prevent strokes by the use of mHealth devices is mainly driven by 2 factors. First, as seen in Table 5, the incidence of prevented strokes tends to increase with increasing CHA_2_DS_2_-VASc scores. The higher the risk score, the higher is the incidence of AF. More patients with AF provide a higher chance to detect AF by using mHealth devices, and thus, initiated anticoagulation therapy will most likely reduce the number of strokes. However, here again, this effect is not seen in groups with a low risk for stroke. The second factor influencing the number of prevented strokes is the ECG confirmation rate. The higher the predictive value of the device is, the more number of cases of AF can be confirmed, and the more strokes might be prevented. If the device diagnosis is more reliable, more cases of AF can be detected and the risk for stroke can be reduced by subsequent therapy. The effects of higher risk scores and high device ECG confirmation rate are even higher in prevented fatal strokes. Nevertheless, there is also no clear effect in low-risk patient groups.

### Sensitivity Analysis

Based on the simulation, a sensitivity analysis was conducted for values of device sensitivity (86%, 93%, and 100%) as well as device false-positive AF detection rate (0.2%, 1%, and 5%) (Table 6 and Table 7). For sensitivity analysis, the confirmation of the mHealth diagnosis was determined to be 75%.

Device accuracy in terms of device sensitivity and device false-positive rate had little impact on the costs per patient but it had big impact on the number of fatal strokes. A higher device sensitivity leads to a higher number of prevented fatal strokes. In terms of the device false-positive rate, a higher value had little impact on costs per patient and the number of strokes. Nevertheless, it should be considered that high false device–positive rates frighten patients and lead to more frequent physician-patient interactions, which are a burden for the health care system.

**Table 6:** Sensitivity analysis. The values were changed to 86% and 100%; 93% was the standard case.

	Device sensitivity								
	86%			93%^a^			100%		
CHA_2_DS_2_-VASc score^b^	Average costs per patient (in €)^c^	Total number of strokes	Number of fatal strokes	Average costs per patient (in €)	Total number of strokes	Number of fatal strokes	Average costs per patient (in €)	Total number of strokes	Number of fatal strokes
1	1275	515	175	1308	558	210	1326	586	209
2	2794	2362	868	2847	2406	821	2816	2312	792
3	3908	3458	1239	3887	3395	1215	3912	3446	1234
4	5445	5111	1861	5380	4994	1790	5456	4986	1728
5	7504	7450	2742	7483	7444	2693	7466	7329	2695
6	9498	9878	3704	9427	9755	3671	9542	9830	3600
7	10,430	10,902	4219	10,646	11,082	4181	10,537	10,814	4106
8	10,831	10,468	4081	10,846	10,374	4079	10,922	10,447	4109
9	12,279	11,696	4715	12,146	11,449	4693	12,129	11,351	4602

^a^Base value.

^b^CHA_2_DS_2_-VASc: Congestive heart failure, Hypertension, Age≥75 years, Diabetes mellitus, Stroke, Vascular disease, Age 65-74 years, Sex category (female).

^c^€1=US $1.12.

**Table 7:** Sensitivity analysis. Values altered for device false-positive atrial fibrillation detection rates.

	Device false-positive rate								
	0.2%^a^			1%			5%		
CHA_2_DS_2_-VASc score^b^	Average costs per patient (in €)^c^	Total number of strokes	Number of fatal strokes	Average costs per patient (in €)	Total number of strokes	Number of fatal strokes	Average costs per patient (in €)	Total number of strokes	Number of fatal strokes
1	1308	558	210	1336	584	207	1342	579	207
2	2847	2406	821	2863	2395	820	2835	2352	789
3	3887	3395	1215	3858	3425	1187	3864	3405	1198
4	5380	4994	1790	5414	5019	1803	5365	4961	1767
5	7483	7444	2693	7526	7452	2735	7447	7254	2626
6	9427	9755	3671	9537	9851	3693	9561	9833	3676
7	10,646	11,082	4181	10,594	10,931	4073	10,650	11,127	4264
8	10,846	10,374	4079	10,923	10,557	4112	10,772	10,305	4026
9	12,146	11,449	4693	12,076	11,373	4691	12,254	11,599	4631

^a^Base value.

^b^CHA_2_DS_2_-VASc: Congestive heart failure, Hypertension, Age≥75 years, Diabetes mellitus, Stroke, Vascular disease, Age 65-74 years, Sex category (female).

^c^€1=US $1.12.

## Discussion

Besides wrist-worn devices, ECG patches, hand-held devices, and apps provide a helpful method to screen for AF [17]. Recent cost-effectiveness analyses of hand-held ECG recorders showed that these devices are likely to be cost-effective in older patient groups [18-20]. Jacobs et al [18] investigated the effect of AF screening with mHealth devices during seasonal influenza vaccination; they found the screening to be cost-effective. A second cost-effectiveness analysis conducted by Aronsson et al [19] showed that 2 weeks of intermittent screening for asymptomatic AF resulted in costs of €4313 per gained quality-adjusted life-year and €6583 per avoided stroke [19]. Levin et al found that screening for silent AF after ischemic stroke in 75-year-old patients leads to decreased costs, extended lives, and improved quality of life [20]. The cost-effectiveness of wrist-worn mHealth devices to detect AF is not yet clarified [17].

The present model is the first to estimate the cost-effectiveness of mHealth interventions by using wrist-worn devices over a long period and assessing the cost-effectiveness of mHealth devices in relation to the CHA_2_DS_2_-VASc score. To assess the health economic effect of mHealth devices, several assumptions and simplifications were integrated in the model. Some costs were excluded. First, in the underlying simulation, indirect costs associated with strokes were not considered. Indirect costs include costs for work loss. Work loss was not considered because no eligible current analysis about those specific costs could have been found. Furthermore, indirect costs incurred by work absences are presumed to be relatively low because strokes mainly occur in older patients who are not working anymore. Second, this simulation was limited to a time period of 10 years. Long-term costs of care and medication were restricted in accordance with the model.

Mean cost values for a visit to the doctor included ordination, consultation, urgent care, telemedical care as well as different types of ECG. Other possible interventions such as international normalized ratio blood test, ultrasound, and radiography [13] were not considered. There were no eligible data for long-term patient care. Thus, subsequent visits were not integrated.

It was implemented that patients with AF receive rivaroxaban because it is the most prescribed NOAC in Germany. Besides rivaroxaban, there are many other pharmaceutical products such as apixaban, dabigatran, warfarin, and phenprocoumon for the treatment of AF. Some patients are not eligible for treatment with NOACs and should take oral anticoagulants in form of vitamin K antagonists (VKAs). Exclusion criteria are, for example, use of mechanical heart valves or moderate as well as severe mitral stenosis [1]. Since the most prescribed VKA in Germany (phenprocoumon: €54.75 per year) is cheaper than rivaroxaban (€1226.40 per year) [15], the estimates in this study are even more conservative. In other studies, the costs for anticoagulation therapy were estimated to be lower. Jacobs et al [18] estimated the costs for NOAC to be €235 in the Netherlands. Aronsson et al [19] suggested the use of apixaban, which resulted in costs of €844 in Sweden.

This simulation is based on published data. However, this published data did not represent a consistent patient pool. Therefore, a special focus was put on the patient characteristics in the underlying studies. The proportion of male and female patients was always near 50%. Patient age as well as other relevant characteristics were represented consistently by the CHA_2_DS_2_-VASc score. A weakness of the simulation was that general mortality in healthy subjects was assumed to be 6%, irrespective of their age.

The stroke incidence in patients with no AF was determined by a division; the stroke incidence of untreated patients with AF was divided by their additional risks for stroke compared to patients with no AF. The most popular study on AF-related stroke risk, the Framingham Study, estimates that the additional risk for stroke in untreated patients with AF compared to that in patients with no AF is 4.8-fold [3]. In this study, this risk was determined to be 2.42-fold according to a meta-analysis by Odutayo et al [2].

The Apple Heart Study showed that only 57% of the patients went to the doctor after receiving an irregular pulse notification [21]. In this simulation, it was modelled that every individual who receives a notification visits the doctor. According to the results, fewer visits to the doctor are related to lower overall costs as well as fewer prevented strokes.

A further problem was to assess the accuracy of the mHealth devices. The assumed accuracy published by Bonomi et al [8] could be overestimated because physical activity, darker skin color, higher body mass index, or male gender may influence the accuracy [22]. With respect to newer devices such as the Apple Watch, more cases of AF can be diagnosed with the aid of ECG recordings in addition to PPG technology. To derive the ratio of AF detected between the groups with and without a device, the findings of a study by Steinhubl et al were used [9]. They investigated the effect of a home-based wearable intervention to detect AF by using ECG patches over a period of 4 weeks. Although Steinhubl et al [9] used ECG patches for a shorter period, their results were integrated in the simulation. Tischer et al [23] found that patients with high CHA_2_DS_2_-VASc scores experienced thromboembolic complications, irrespective of the presence of AF. In these patients, anticoagulation therapy may be initiated, regardless of AF. Thus, particularly in the group with devices, for higher scores, the costs of the prescribed NOACs could be overestimated because some patients would receive anticoagulation therapy, irrespective of AF.

In conclusion, the results of this simulation allow the assessment of the use of mHealth devices in different risk groups. From an economic point of view, the use of these devices in patients with high risk scores increases the costs per patient. With higher risk scores, costs per prevented stroke decrease. Higher device accuracy leads to more stable results. From a patient-oriented perspective, the use of mHealth devices results in reduced number of strokes. More strokes can be prevented if the underlying CHA_2_DS_2_-VASc score is higher. In addition, a high ECG confirmation rate and increased device accuracy lead to more prevented strokes.

This study shows that mHealth devices are a recommendable tool to screen for AF in patients with high CHA_2_DS_2_-VASc scores. The higher the risk for stroke in patients with AF, the more cost-effective are the devices.

## Abbreviations

AF: atrial fibrillation
CHA_2_DS_2_-VASc: Congestive heart failure, Hypertension, Age≥75 years, Diabetes mellitus, Stroke, Vascular disease, Age 65-74 years, Sex category (female)
ECG: electrocardiography
mHealth: mobile health
NOAC: non–vitamin K antagonist
PPG: photoplethysmography
VKA: vitamin K antagonist
